# TasselGAN: An Application of the Generative Adversarial Model for Creating Field-Based Maize Tassel Data

**DOI:** 10.34133/2020/8309605

**Published:** 2020-08-03

**Authors:** Snehal Shete, Srikant Srinivasan, Timothy A. Gonsalves

**Affiliations:** School of Computing and Electrical Engineering, Indian Institute of Technology, Mandi, India

## Abstract

Machine learning-based plant phenotyping systems have enabled high-throughput, non-destructive measurements of plant traits. Tasks such as object detection, segmentation, and localization of plant traits in images taken in field conditions need the machine learning models to be developed on training datasets that contain plant traits amidst varying backgrounds and environmental conditions. However, the datasets available for phenotyping are typically limited in variety and mostly consist of lab-based images in controlled conditions. Here, we present a new method called TasselGAN, using a variant of a deep convolutional generative adversarial network, to synthetically generate images of maize tassels against sky backgrounds. Both foreground tassel images and background sky images are generated separately and merged together to form artificial field-based maize tassel data to aid the training of machine learning models, where there is a paucity of field-based data. The effectiveness of the proposed method is demonstrated using quantitative and perceptual qualitative experiments.

## 1. Introduction

Field-based plant phenotyping is a process wherein the desired plant traits are studied in the plant's natural environment throughout its growth cycle. Observations of the desired traits are carried out using various sensors including cameras, and the amount of data to be processed can be very large. Various imaging techniques and machine learning algorithms have enabled the development of high-throughput image-based automated phenotyping methods. However, uncontrolled environmental variations pose a major challenge to such methods [1–3].

According to the plant phenotyping survey conducted by the international plant phenotyping network [4], the need for high-throughput field phenotyping systems has increased and the crops of interest are largely wheat and maize. Maize, being a highly produced crop, is extensively phenotyped for improving its yield and desired traits [1, 3, 5]. One of the important traits of maize plants is the tassel (male flower of maize) architecture as tassels play a major role in the pollination process. However, datasets available for high-throughput machine learning-based maize tassel phenotyping are rather limited and are either lab-based [5] or do not have a detailed tassel view [1]. This is a concern considering that the performance of machine learning algorithms relies on a comprehensive training dataset.

To deal with the problem of limited training data, techniques for data augmentation [6, 7] and other approaches such as weak supervision and active learning-based algorithms are used [8, 9]. In weak supervision, one of the following three approaches is used to label the unlabelled data: less precise annotations by nonexperts, higher-level labelling by subject experts, and use of existing pretrained models or alternate datasets for labelling. These weak labels are helpful in increasing the efficiency of hand-labelling data [8]. In active learning, the most valuable data from the unlabelled data pool are presented to the subject experts or human oracle for labelling [8, 9].

In order to increase the image data, traditionally augmentation techniques such as geometric transforms, colour, or brightness changes have been routinely employed [6]. However, these techniques may produce limited variations of the training data samples. On the other hand, generative models [7] learn the distribution of training data in order to produce new, unseen examples that are similar to training data samples. An excellent recent survey of generative methods, in particular, evolution and evaluation of generative adversarial network (GAN) models, is presented in [7]. The use of generative networks is demonstrated in advanced active learning methods such as [10–13].

Some recent works have also conducted weak supervision or active learning-based methods and the task of synthesizing plant data, as follows.

 
*Weak Supervision or Active Learning for Labeling Plant Data*. In [14], a weak supervision method is used in sorghum head detection and counting using UAV images. Here, human supervision is used for checking bounding box labels in images. The method in [15] uses active learning for two-level annotations of panicles in the cereal crops wheat and sorghum. In the first level, weak annotations involving the position marking center of panicle object are obtained. Using a subset of weakly labelled data, further annotations at bounding box level are obtained. Another work [16] uses weak supervision for land cover mapping.
 
*Generation of Synthetic Plant Data.* In the case of plant data, synthetic generation is reported in [17–19] for lab-based Arabidopsis data. The motivation behind these works is tackling the task of leaf segmentation and counting. Arabidopsis Rosette Image Generator (through) Adversarial Network (ARIGAN) [17] uses a conditional GAN-based approach [20] to generate Arabidopsis samples where the condition is a number of leaves to be generated. In [19], the Lindenmayer system (L-system) [21] is used for generating plant leaf data. The L-system model has parameters such as leaf shape and inclination angle. A software tool Blender is used for creating synthetic data in [18]. In [22], synthetic images of the capsicum plant having natural background are generated using software PlantFactory 2015 Studio.

Summary of contributions. In continuance of the above efforts towards synthetic generation for plant data and the need to extend it to more complicated phenotyping tasks in field conditions, we propose TasselGAN, a method for generation of field-based maize tassel dataset. The generated dataset has images with maize tassels against a sky background. Each image contains a single tassel. The foreground maize tassels and sky background data are generated separately, using a variant of deep convolutional generative adversarial network (DC-GAN) [23], and merged together to form output images. The masks of generated maize tassels, which can be used for segmentation problem, are also created. We also provide qualitative and visual quality results of the generated data.

## 2. Materials and Methods

In this section, we discuss in detail the proposed maize tassel dataset generation method, TasselGAN, which consists of maize tassel data and sky data generation, and then merging the two to form field-like dataset. Natural scenes are complex, and hence, to simplify the synthesis problem, foreground and background are separately generated. This approach is used in [24–26].

We begin with a brief overview of GAN. Recent techniques that use deep learning for generating data are variational autoencoder (VAE) [27] and DC-GAN [23]. However, GANs have been shown to produce visually more appealing results than VAE [7]. Hence, in our method, we have used modified DC-GAN architectures for separately generating maize tassels and sky background data. We have trained our networks using a NVIDIA GTX 1080 Ti GPU.

### 2.1. Generative Adversarial Models

The basic idea proposed in GAN [28] is designed around two networks named the generator and the discriminator, which compete against each other. The generator tries to mimic the distribution of the training data and generate samples, whereas the discriminator tries to identify if a sample given to it is from training data or from the generated data. The training process in GANs is inspired by the two-player mini-max zero-sum game [29] between the generator and the discriminator. If the generator's loss decreases, resulting in a better quality of artificially generated samples, the discriminator's loss increases due to difficulty in classification between real and generated samples. The discriminator is trained first followed by the generator. Backpropagation through both these networks enables the generator to update its weights to make better output samples.

**Figure 1:**
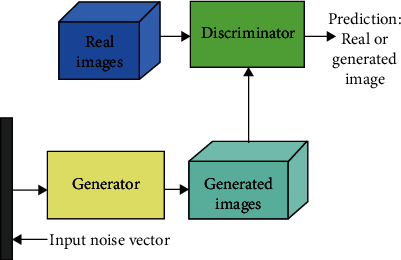
Basic block diagram of the generative adversarial network (GAN).

Figure 1 shows the basic block diagram of GAN. The detailed mathematical explanation regarding the loss computations can be found in [28]. The DC-GAN approach [23] implements GAN using convolutional neural networks.

### 2.2. Maize Tassel Generation

For generating maize tassels, we have used images from a dataset captured in an indoor lab [5]. The images in this dataset have a resolution of 4000 × 6000. The detailed information of maize plant material and the camera set-up used for capturing tassel images is provided in [5].

The training dataset consisting of 1224 images is then constructed after cropping, segmenting, and resizing the original data. The central part of the images having the tassel is first cropped, and the size of the cropped images is 2512 × 3408. This size is selected based on the maximum width and height values of the tassels used in our training set.

**Figure 2:**
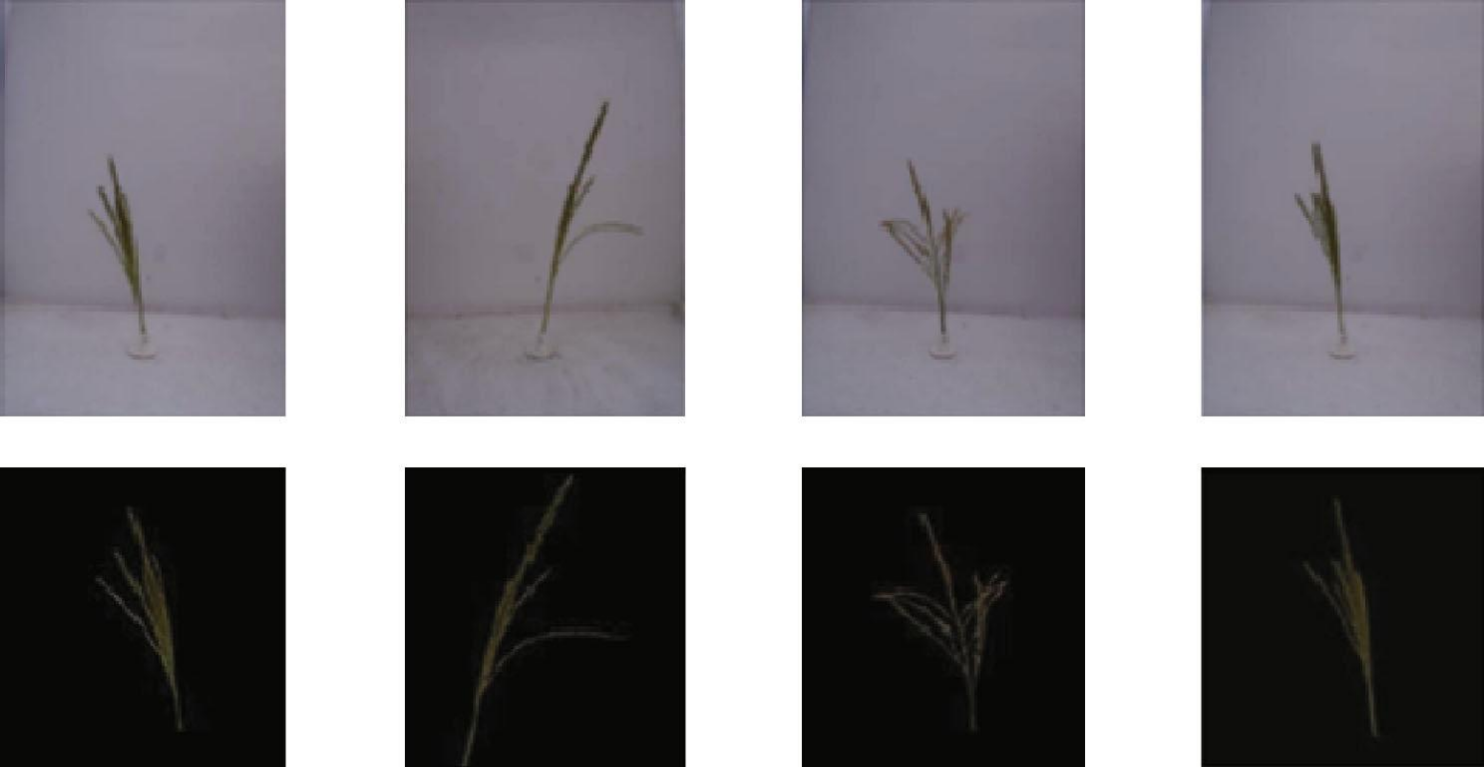
Maize tassel training dataset samples. First row: samples from tassel training data [5]. Second row: samples used for training GAN after cropping, segmenting, and resizing.

For segmentation, the tassel images are first converted in YCbCr colour format. Otsu thresholding is then applied to the Cb colour channel of the tassel images for segmenting the tassel. The segmentation step is necessary since we are interested in creating a field-based dataset, with the tassel overlaid on a sky background. The images are then resized to 128 × 128 resolution.  Figure 2 shows example images used for training in tassel generation.

While using the vanilla DC-GAN architecture [23], we have observed that discriminator loss was becoming zero within a few cycles and, hence, the generator weights were not updated. In this case, the generator was unable to produce good output images. However, the removal of batch normalization layers from both the generator and discriminator networks enabled the training process to update the generator and discriminator weights. The generator was able to produce 64 × 64 resolution images after this modification.

As generated images having resolution 64 × 64 are too small for any practical application, we have generated 128 × 128 images by adding one more layer to the above DC-GAN architecture. Here, we encountered a degradation problem [30]. The DC-GAN model designed for 128 × 128 resolution was not able to learn colours of input maize data against a uniform black background.

**Figure 3:**
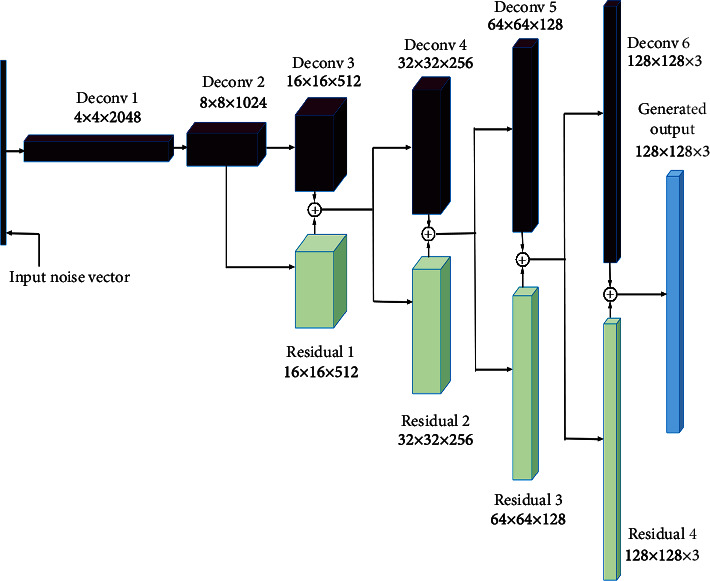
Modified generator architecture for maize tassel generation.

In order to overcome the degradation problem, we added residual blocks, as presented in ResNet architecture [30], to the generator network of our DC-GAN variant.  Figure 3 shows the modified generator architecture used for maize tassel generation in our work. We have empirically found that it was sufficient to add residual blocks to 16 × 16, 32 × 32, 64 × 64, and 128 × 128 deconvolution (Deconv) layers. Here, the Deconv layers indicate transposed convolution followed by the ReLu layer, except for the last layer where *tanh* activation is used. The input training image data in the DC-GAN [23] model is normalized in the range [−1,1] before giving to the discriminator. Hence, in the last Deconv layer of the generator, *tanh* activation [31] is utilized to set the generated data range to [−1,1].

#### 2.2.1. Residual Computation

**Figure 4:**
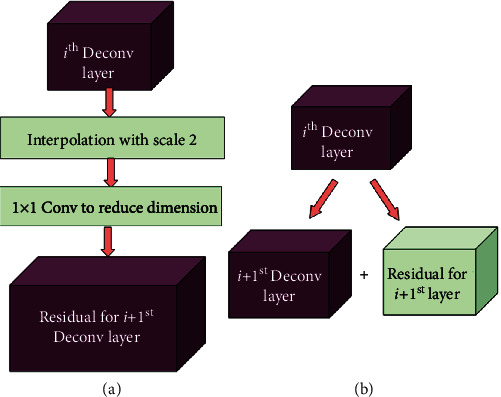
Computing and adding residual. (a) Computation of residual using the *i*^th^ Deconv layer. (b) Adding residual to the *i* + 1^st^ Deconv layer.

The consecutive Deconv layers in the generator network have the resolution increased by a factor of 2, and the number of output channels is reduced. Hence, in order to match the resolution of residual to the next, say *i* + 1^st^, Deconv layer, we have upscaled the current *i*^th^ Deconv layer feature maps by a factor of 2 using interpolation. For matching the number of output channels of residual to that of the *i* + 1^st^ Deconv layer, the upscaled feature maps, now having the number of output channels of the *i*^th^ Deconv layer, are further subjected to 1 × 1 convolution layer with zero padding and 1 stride.  Figure 4 shows the computation of residual using the input *i*^th^ Deconv layer to be added to the *i* + 1^st^ Deconv layer and addition of the *i*+1^st^ Deconv layer and residual.

#### 2.2.2. Variations to the Generator Network

A summary of the variations that we have made to the generator network architecture, to suit our problem of generating maize tassel data, include (a) removal of batch normalization layers and (b) addition of residual to 16 × 16, 32 × 32, 64 × 64, and 128 × 128 Deconv layers. The detailed observations of these modifications are provided in the ablation study section.

In GAN, the last layer of the generator network is mapped to RGB colour channels using convolution. It may be noted that in the case images taken by digital cameras, spectral response functions are used to form a RGB image. The weights used for forming pixel values using these functions are different than the learned weights by a generator in GAN. Hence, the colours of GAN-generated images appear different than the real images [32–34]. In our case, the generated foreground maize tassel images were brighter than the training tassel images. Hence, postprocessing was done to reduce their luminance.

### 2.3. Sky Background Generation

In the case of generation of sky background patches, we have used images from two datasets, namely, Singapore Whole sky IMaging SEGmentation Database (SWIMSEG) [35] and HYTA dataset [36]. SWIMSEG dataset has images with resolution 600 × 600, and HYTA dataset has images with resolution 300 × 300 and above.

**Figure 5:**
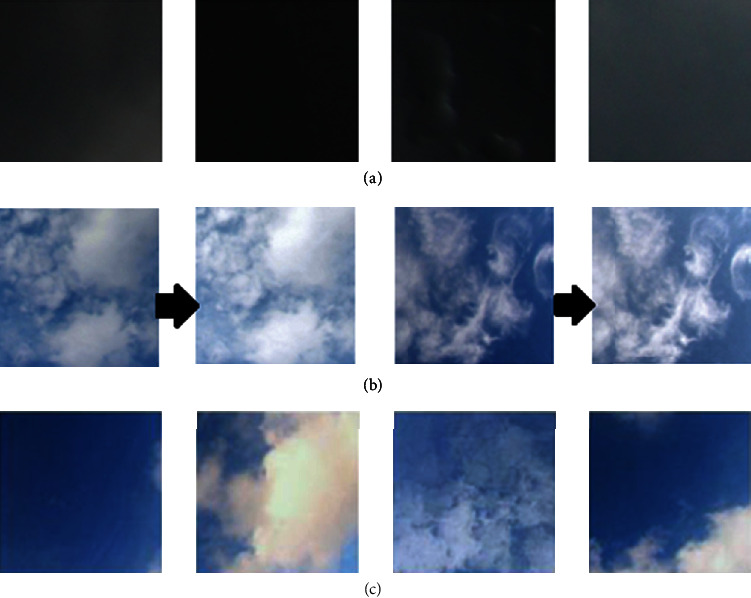
Training data for sky patch generation: (a) example of dark sky patches in SWIMSEG Database [35] which were removed; (b) first pair of images: example of augmentation using saturation and luminance change, second pair of images: example of augmentation using saturation change; (c) example of sky patches in SWIMSEG Database [35] used as training data samples.

In the SWIMSEG dataset, there is a category of thick dark sky images (Figure 5(a)). These darker images were removed from our training dataset because of the following reasons: the dark images of sky background were not included in our dataset since the foreground tassel data were obtained under well-lit conditions and would not form realistic images when merged with the dark background. Also, RGB-based imaging is typically carried out under bright conditions.

The sky data is augmented with a few images after processing some of the existing images in SWIMSEG data. The images were first converted into HSV colour format. Then, images obtained, after scaling saturation by a value of 0.6 and in some cases further scaling the luminance by 1.3, were augmented in our training data. These scaling factors were found empirically. Figure 5(b) shows an example of images created for augmentation. Figure 5(c) shows an example images used for training for our sky background generation. Our training dataset has 1100 images.

In this case, we have followed the DC-GAN model similar to maize tassel generation, with the batch normalization layers removed from generator and discriminator networks. Our sky training images do not have any particular structure, and the colours in the images are majorly variations of blue and white. Hence, removing batch normalization did not have any adverse effects.

Since we did not see any degradation while generating 128 × 128 sky patch images, there was no need for residual addition in generator network. Hence, the model used for generating sky background is basically DC-GAN architecture [23] without batch normalization layers and with an additional layer for 128 × 128 resolution, in both the generator and discriminator networks.

### 2.4. Synthetic Maize Tassel Data against Sky Background

The synthetic field-based maize tassel data was created by merging the foreground maize tassels and background sky patches. First, we upscaled the maize tassels and sky patches by a factor of 2, using bicubic interpolation. For creating the tassel masks, we converted the generated tassel patch into YCbCr format, which segregates luminance (Y), blue chrominance (Cb), and red chrominance (Cr) in three channels. Thresholding was then applied to the Y channel owing to the maximum separation between the maize tassel and background pixel values. The maize tassels and sky patches are then merged using

(1) $$\begin{array}{c} I_{m}=\text{mask}*I_{f}+\left(1-\text{mask}\right)*I_{b}, \end{array}$$

where *I*_*m*_, *I*_*f*_, and *I*_*b*_ are merged image, foreground tassel image, and background sky patch.

In order to get seamless merging, we have computed weighted addition at all edge pixels and its left and right neighbouring pixels in a row, of image *I*_*m*_.

(2) $$\begin{array}{c} I_{\text{medge}}=0.5*I_{\text{medgeL}}+0.5*I_{\text{medgeR}},\\ I_{\text{medgeL}}=0.75*I_{\text{medgeL}}+0.25*I_{\text{medgeR}},\\ I_{\text{medgeR}}=0.25*I_{\text{medgeL}}+0.75*I_{\text{medgeR}}, \end{array}$$

where *I*_medge_, *I*_medgeL_, and *I*_medgeR_ are edge pixels in *I*_*m*_ and its left and right neighbouring pixels, respectively.

**Figure 6:**
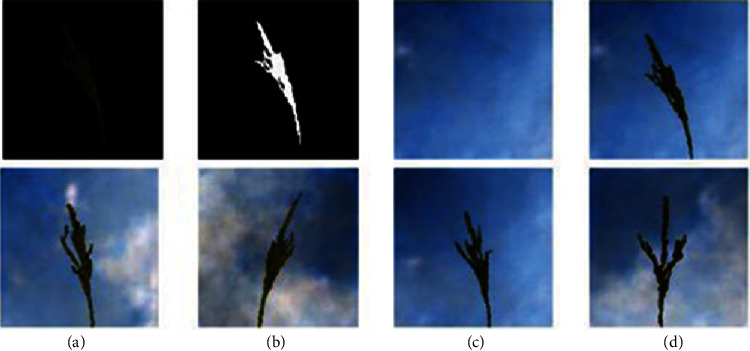
Steps for synthetic generation of field-based maize tassel data: first row: (a) generated tassel after luminance correction, (b) tassel mask, (c) generated sky patch, and (d) output image after shifting and merging tassel with a sky background. Second row: samples from our synthetic field-based maize tassel data.

The merged image was scaled down by a factor of 2. The first row of Figure 6 shows the steps followed in the generation of this data and some of the sample images.

## 3. Results

### 3.1. Maize Tassel and Sky Background Generation and Merging

In this section, we present the results of our generative models and field-based maize tassel images after merging outputs of these models.

The variant of DC-GAN used for generating maize tassels was trained for 2500 epochs at a learning rate of 1.5 × 10^−5^. We have empirically observed that if residual addition in our model continues after 1000 epochs, noise starts appearing in the generated tassels. Hence, we have trained our model with residual addition till 1000 epochs and disabled residual addition for the rest of the training cycles. The training time for 2500 epochs for tassel generation was found to be 6 hrs and 36 mins.

For generating sky background, the model is trained for 600 epochs at a learning rate of 2 × 10^−5^. The other parameters like adam optimizer and cross-entropy loss function in both our models are the same as reported in [23]. Training time for 600 epochs for the sky generation was found to be 6 hrs and 25 mins.

**Figure 7:**
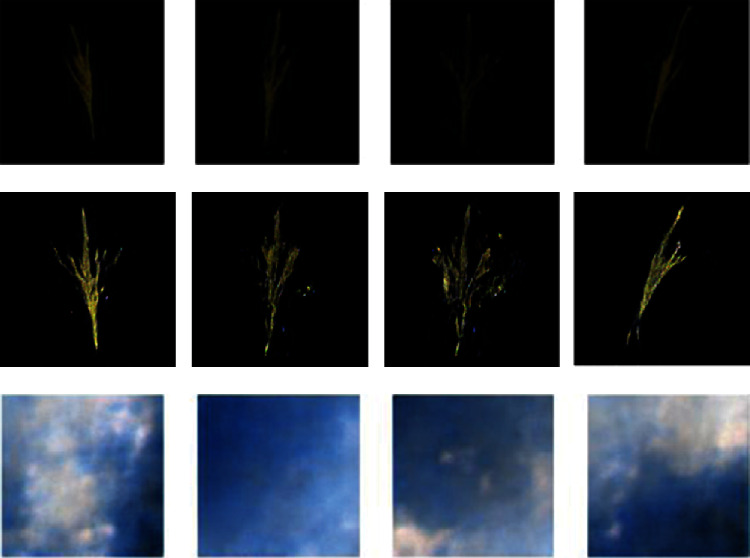
Generation results: first row: generated maize tassel samples after 2500 epochs and postprocessing. Second row: example of noisy generated maize tassel samples. Bottom row: generated sky patches after 600 epochs.

The first row of Figure 7 shows the maize tassel generation results. In the generated maize tassel images, the overall structure of tassels is well preserved. Also, one can see details such as separated branches, different orientation, and shapes of tassels. However, we found that for around 5% of the cases, the generated images are noisy. This is shown in the second row of Figure 7. The bottom row of Figure 7 shows sky background generation samples which have a natural sky-cloud texture. Sample images from the synthetic field-based maize tassel data are presented in the second row of Figure 6.

### 3.2. Ablation Study

In order to understand the contribution of different layers of our model in the generation of maize tassels and sky patches, we have performed an ablation study [37, 38], which involves the stripping away of specific layers to gauge their effect on the output. We have analysed the training losses and feature maps of the discriminator in the GAN models. The below subsections give the details of this study.

#### 3.2.1. Effect of Removing Batch Normalization Layers in Maize Tassel Generation

The original DC-GAN design proposed in [23] has batch normalization layers in both the generator and discriminator architectures. The batch normalization layer stabilizes input features to a layer by restricting their distribution to zero mean and unit variance. The paper states that batch normalization layers avert mode collapse scenarios in which the generator produces similar looking samples. The first convolution layer in the discriminator and the last deconvolution layer in the generator do not have batch normalization layers. This is because the batch normalization layers when used in all layers resulted in model instability [23].

**Figure 8:**
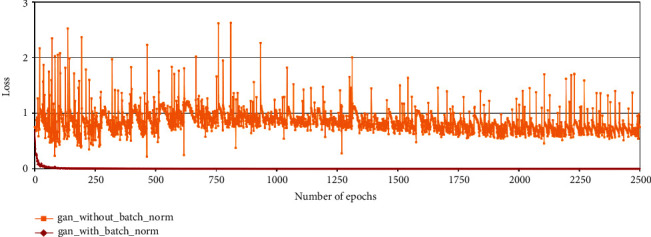
Discriminator losses in the DC-GAN model, used for maize tassel generation, with and without batch normalization layers: red curve shows discriminator loss with batch normalization layers and yellow curve shows discriminator loss without batch normalization layers.

When the original DC-GAN is used in the tassel generation task, the discriminator loss becomes zero within a few cycles. The red curve in Figure 8 shows this behaviour.

**Figure 9:**
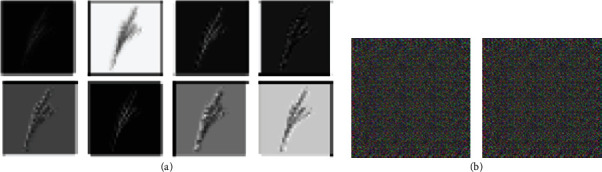
Selected feature maps of discriminator and output images of a generator in the GAN model having batch normalization layers: (a) feature maps of the second Conv layer, (b) generated images (the feature maps are scaled up for visualization purpose).

We have analysed feature maps of discriminator network in this DC-GAN model after training it on our tassel training data.  Figure 9 shows sample feature maps of discriminator and output images of the generator using DC-GAN architecture with batch normalization layers. Here, we can see that the discriminator has learned some features in the first few epochs. However, since the discriminator converged very early and did not update weights, the generator did not learn and, at this stage, was still producing noisy images, which the discriminator easily identified as fake.

In GANs, since the discriminator and generator are competing against each other, the better the discriminator is trained, the better is the feedback given to the generator using backpropagation. Also if the generator gets more training iterations, it will be able to learn the features that should be present in the generated images. Thus, keeping the batch normalization layers, we experimented with reducing the learning rate and giving more training chances per epoch to the generator. However, this did not result in the generation of better samples.

**Figure 10:**
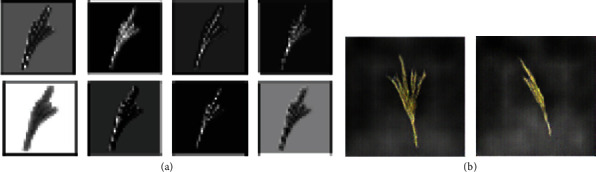
Selected feature maps of discriminator and output images of a generator in the GAN model after removing batch normalization layers: (a) feature maps of the second Conv layer, (b) generated images (the feature maps are scaled up for visualization purpose).

We then considered the fact that batch normalization layers also help in faster loss convergence [39]. Hence, in order to delay the convergence of the discriminator and generator, we experimented with removing batch normalization layers from both the discriminator and the generator. The yellow curve in Figure 8 shows that after removing batch normalization, the discriminator loss did not converge early, and hence, the generator was able to learn and generate better samples.  Figure 10 shows sample feature maps of discriminator and output images of the generator using DC-GAN architecture without batch normalization layers.

Also in the tassel generation training images that we have used, the foreground tassel data is very less as compared to the black background (bottom row of Figure 2). The colour information does not show many changes and even though there are some variations in terms of structure, these variations are not significant. Hence, the removal of batch normalization did not negatively affect the model's performance in our case.

#### 3.2.2. Effect of Adding Residual Layers

**Figure 11:**
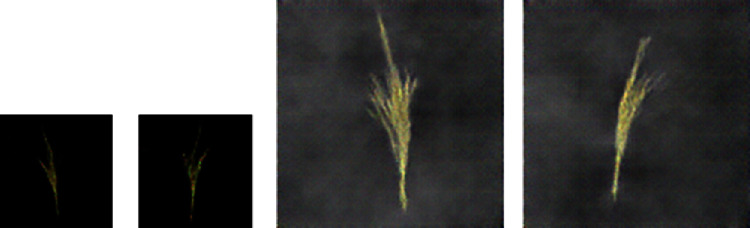
Degradation problem. First two 64 × 64 images generated using 5-layered GAN and third and fourth 128 × 128 images generated using 6-layered GAN (he images are scaled up for visualization purpose).

We have encountered the degradation problem while generating 128 × 128 resolution images, which occurs due to higher training error when more layers are added to a smaller network [30]. Here, the smaller network having 5 Deconv layers (64 × 64 resolution) was performing better than the network with 6 Deconv layers (128 × 128 resolution).  Figure 11 shows output images of both these networks, where we can clearly see degradation in 128 × 128 images.

**Figure 12:**
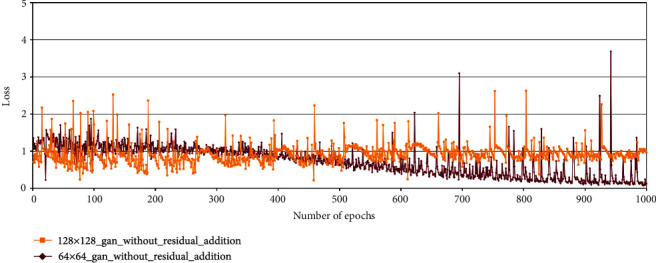
Discriminator losses in the DC-GAN model after removing batch normalization layers. Maroon curve: 64 × 64 model and dark yellow curve: 128 × 128 model.

In the case of generation of 64 × 64 maize tassel images, after removing batch normalization layers from the original DC-GAN model, the discriminator loss converges at around 1000 epochs during training (maroon curve in Figure 12). On the other hand, the training error in the discriminator is higher while generating 128 × 128 maize tassel images (dark yellow curve in Figure 12).

We have used a residual addition approach [30] in the generator network of DC-GAN. After residual addition, the generator is able to produce images that are fairly similar to the training images. Here, the discriminator loss decreased after the addition of residuals in the generator. Again, this is because of the adversarial nature of GAN where as the generator is able to produce better images, the discriminator is able to identify the real and artificial images better.

**Figure 13:**
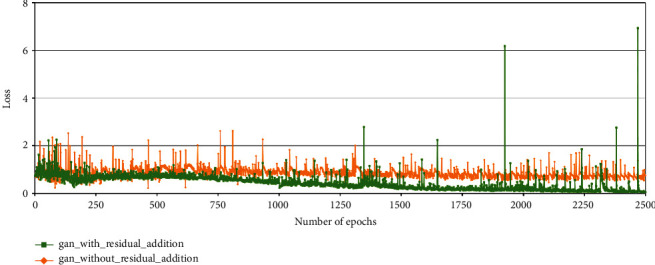
Discriminator losses in the DC-GAN model used for maize tassel generation with and without residual addition. Yellow curve: discriminator loss without residual addition, green curve: discriminator loss with residual addition.

**Figure 14:**
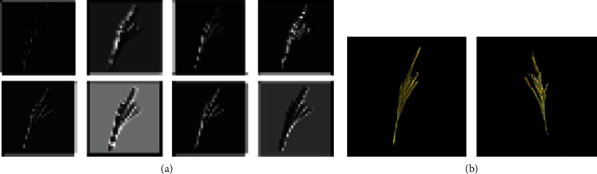
Selected feature maps of discriminator and output images of a generator in the final GAN model: (a) feature maps of the second Conv layer, (b) generated images (the feature maps are scaled up for visualization purpose).

The yellow and green curves in Figure 13 show discriminator losses without and with residual addition, respectively.  Figure 14 shows the feature maps and generated output images of our variant of DC-GAN after removal of batch normalization layers from the discriminator and generator network and after the addition of residual layers in the generator.

### 3.3. Qualitative Results of Synthetic Field-Based Maize Tassel Images

We have evaluated the perceptual quality of the synthetic field-based maize tassel images by using two tests. In the first test, we have created a dataset consisting of field-based maize tassel images made by merging training (real) tassel images and training (real) sky patch images. We have randomly selected 51 of such merged images from these and called them “real images.” We have selected 49 images of synthetic field-based maize tassel data created using the method described in Section 2.4 and called them “generated images.”

**Figure 15:**
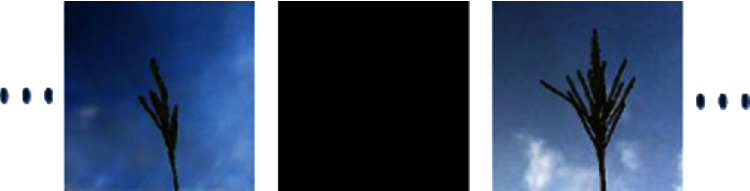
Experimental set-up of real vs. generated image identification.

These 100 images were then randomly displayed to human participants. We asked 10 human participants, 7 students/staff working on botanical and farm-related projects and 3 computer science students, to identify each image as real or generated. Each image was displayed for 5 seconds, and a black image was then displayed for 2 seconds, in between consecutive images, in order to remove the influence of the earlier image.  Figure 15 shows the experimental set-up of this test. The participants were supposed to make a choice about an image, real or generated, within 5 seconds when that image was displayed on the screen. We collected a total of 1000 observations from 10 participants.

The criterion of selection was the naturalness of the image, and participants normally could distinguish between real and generated images. In this experiment, participants form and evolve their opinions of annotating an image as real or generated, as the images are progressively displayed to them. Ideally, when the generated images are a very close match with the real images, all the entries in Table 1 should be 50%.

In our case, 39.38% of the generated images are sufficiently similar to the real ones, causing them to be classified as real. Interestingly, the perceptual ambiguity between real and generated images also results in the annotation of 27.64% real images as generated. As the area of visual data generation is relatively new, such performance is considered to be encouraging even in well-known early works on GAN. For instance, in the work on video generation [24], the authors report that in 16-18% trials, generated videos are identified as realistic. Considering that ours is an early work on tassel image generation, we believe the results of this experiment too are reasonably good.

In the second test, we constructed 50 pairs of real and generated field-based maize tassel images. We asked the participants to rate these pairs for image similarity on the scale of 1 (image not similar) to 5 (images highly similar). It should be noted that the images generated using GAN do not have a one-to-one correspondence with the training images. However, since the GAN learns the distribution of the training images, often there is a sharp similarity between the training and generated images and these can be used to construct image pairs.

**Figure 16:**
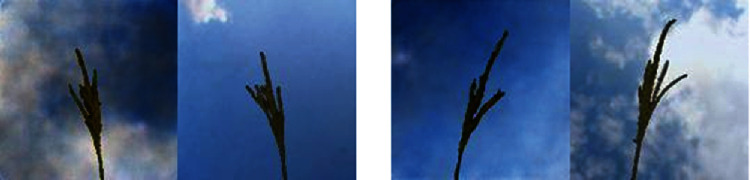
Examples from image similarity experiment.

We again collected 500 observations from the same 10 participants. The average image similarity rating from these observations is 3 indicating the reasonable realistic visual quality of our generated field-based tassel.  Figure 16 shows example pairs of generated and real maize tassels used in this experiment.

#### 3.3.1. Inter-annotator Agreement

For the above two experiments, we have computed the inter-annotator agreement score using a method proposed in [40]. In this method, a lower bound on error, i.e., difference of opinion between annotators, is computed. For *K* number of annotators and *N* number of items to be annotated, this error bound is defined as

(3) $$\begin{array}{c} e\geq \frac{1}{KN}\overset{N}{\underset{i=1}{\sum }}\underset{j}{\min}\left\{K-X\left(i,j\right)\right\}, \end{array}$$

where *X*(*i*, *j*) is the number of annotators that have labelled *i*^th^ item with label *j*. An error of value 0 indicates no disagreements between the annotators while 1 indicates complete disagreements.

In the first perceptual quality test above, the images are annotated into two classes, real and generated. The inter-annotator error in this case, for 10 annotators and 100 images, is 0.302. For each participant, we computed the expert level based on the number of real images that are classified correctly by the participant. For the top 5 annotators, indicated by this level, the inter-annotator error is 0.222.

In the case of the second test, we computed inter-annotator error based on two categories: image similarity rating less than 3 and greater than or equal to 3. The inter-annotator error for 10 annotators and 50 pairs of images is 0.304. For the top 5 annotators, the inter-annotator error is 0.288.

### 3.4. Quantitative Results of Generated Maize Tassels

#### 3.4.1. Comparing Widths and Heights of Training and Generated Tassels

**Figure 17:**
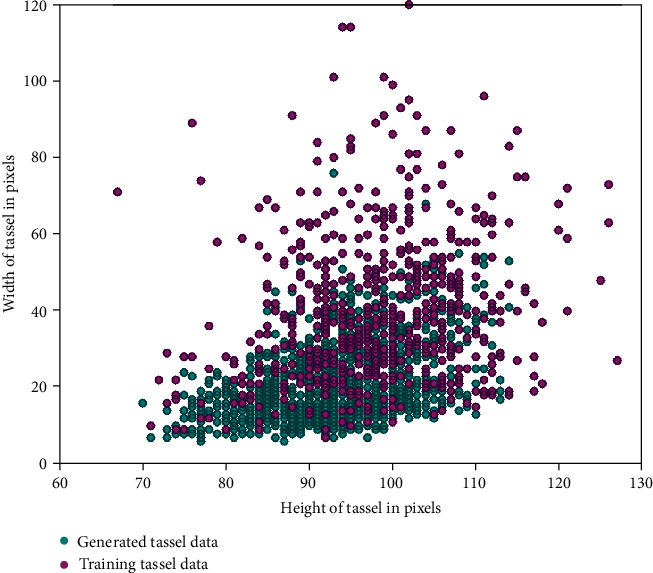
Width vs. height scatter plot showing training and generated tassel data.

We have computed widths and heights of generated and training maize tassels on the bounding box level. The training and generated tassels were first represented in YCbCr format, and then, the Y channel was subjected to Otsu thresholding. Bounding box coordinates were computed using thresholded images.  Figure 17 is a scatter plot of width against the height of tassels, showing training and generated maize tassel data. The plot has 1224 generated and training maize tassels. The blue-coloured markers represent generated tassel data, and magenta-coloured markers represent training tassel data.

The interpretation of the scatter plot is as follows:

- Tassel height comparison: along the *x*-axis, the generated tassel height values (blue markers) show almost the same spread as that of the training tassel height values (magenta-coloured markers). The standard deviations of heights of training and generated data are 9.54 and 8.19, respectively.
- Tassel width comparison: along the *y*-axis, the generated tassel width values fall within the range of the training tassel width values. However, tassels having larger width are not generated. Therefore, the standard deviations of training and generated data widths are 19.21 and 10.23, respectively. This might be because only a small part (14%) of the training tassels has widths of more than 60 pixels. For the major part (84%) of training tassel data, width variations are captured in generated tassel widths.
- These observations suggest that the variations in widths and heights of training maize tassels are replicated faithfully in generated maize tassels.
- Further, this data can be used for developing algorithms for trait extraction such as tassel length, width of tassel, and number of branches.

#### 3.4.2. Structural Similarity (SSIM) Index Computation for Training and Generated Tassels

The Structural Similarity (SSIM) index [41] is a metric indicating the similarity between two images. SSIM takes into account luminance, contrast, and structural comparisons between two images for computing a similarity score. SSIM 0 indicates no similarity, and 1 indicates exact similarity.

We have computed SSIM [41] between 1224 pairs of generated and training maize tassels. For each of the 1224 training tassels, a generated tassel is randomly selected and SSIM is measured between the selected pair. We compute the average SSIM score across 1224 pairs of images. The final SSIM score is the average of 3 such measurements. The SSIM score between training and generated tassels is 0.8429 which indicates a high level of similarity. The SSIM score computed for merged real and generated image pairs created in the perceptual quality experiment is 0.8527.

In the case of GAN-generated images, an intraclass SSIM score is used to show the variability of the data [42]. We computed the SSIM score for each of the tassel by comparing it against a randomly selected tassel from the same dataset. The SSIM score is obtained by using the computations described above. In our case, the intraclass SSIM score for training and generated tassel is 0.8447 and 0.8927, respectively. The high intraclass SSIM score is due to the fact that tassel position in all the images is at the center of the image and the approximate tassel structure is similar to central spike and branches. Also, the tassels have a black background which contributes to this high SSIM score.

#### 3.4.3. Improved Classification Using Generated Tassels

The usability of the generated tassel data is demonstrated using a simple experiment of tassel classification. The classifier used in this case is the K-Nearest Neighbours (KNN) classifier. The features used for classification are widths and heights of tassels computed in 3.4.1.

While preparing the training dataset, every 7^th^ sample out of 1224 training maize tassels was selected. Depending on the spread of the tassels, these 175 samples were manually labelled into two classes as closed_small_tassels or open_wide_tassels. We have 115 of small tassels and 60 wide tassels.

In the case of the generated data, we labelled 141 small tassels and 34 wide tassels. In this case, we first selected all the wider tassels that are generated, and then from the rest of generated tassels, every 5^th^ sample was selected. This data was used for appending the above training data in the classification task.

We used the Scikit-learn library, machine learning library for python for performing this experiment. First, the training dataset consisting of 175 images was divided into 50% training and testing samples using the train test split function in Scikit. The generated data was appended gradually, in steps of 25% samples, to this training data. The number of neighbour parameter selected for KNN classification was 5.

The result of this toy experiment shows an increase in testing accuracy (Table 2). The testing accuracy increases by 3% and further by 1% when the training data is appended with 25% and 50% of generated data. In the last case when 75% of data is appended to, there is no additional improvement in the accuracy.

We acknowledge that the synthesis of maize tassels is a relatively new problem in the maize phenotyping area, and this is an early effort to address it. This work can pave the way for more improved generations, in order to use it in the supervised tasks related to maize phenotyping.

### 3.5. Generation of Maize Tassels Using Merged Tassel and Sky Training Data

**Figure 18:**
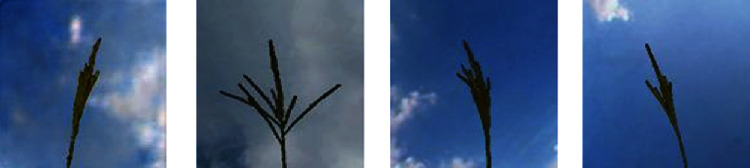
Training data: maize tassels generated using merged tassel and sky data.

We experimented with generating maize tassel images against sky background using images formed by merging the maize tassels and sky patches from the training data.  Figure 18 shows example images from this training data.

**Figure 19:**
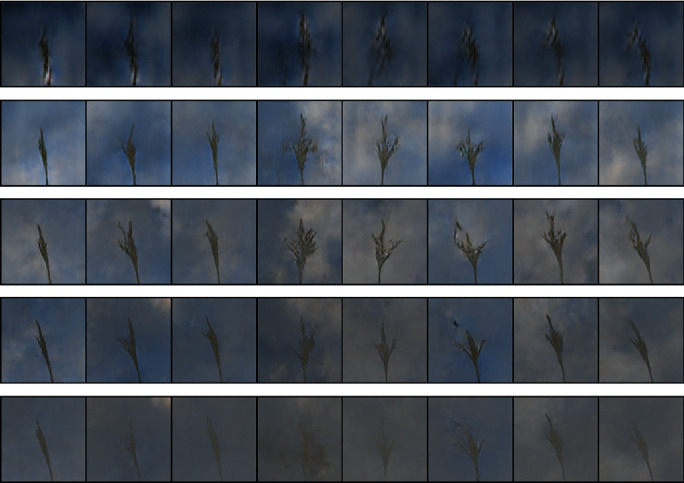
Progress of maize tassels generated using merged tassel and sky training data: first, second, third, and fourth rows depict generation after 300, 500, 1000, 1500, and 1550 epochs.

We used 1224 training images of resolution 128 × 128, and the model used for generation is the DC-GAN model without batch normalization.  Figure 19 shows the progress of this generation model. We observed that after 500 epochs the sky part starts converging, and the foreground tassels start converging around 1500 epochs. However, in this case, the colour of both sky and tassel parts has less contrast. Further, we saw that the generation starts degrading after 1500 epochs.

## 4. Conclusion

We have developed TasselGAN, a new method to create synthetic image data having maize tassels against a sky background. TasselGAN incorporates variations in DC-GAN to generate maize tassels and sky patches, which are then merged to create a field-based imaging data. Our method, TasselGAN, produces 128 × 128 resolution images.

The widths and heights of generated and training tassel data were compared at the bounding box level, showing that the generated tassels have captured the variations of training tassel data. Visual quality and similarity analysis, using human participants, also yielded encouraging results, which indicate that the generated images are indeed realistic. The 128 × 128 resolution images are sufficient to measure traits such as branch numbers and tassel length. To our knowledge, this is the first attempt to create a synthetic field-based phenotyping data for maize tassels. In the future, we plan to improve the resolution and the visual appeal of images and explore the generation of images having different environmental conditions due to the time of day and seasonal effects.

## Figures and Tables

**Table 1:** Results: real vs. generated image identification.

	True real	True generated
Perceived real	72.36%	39.38%
Perceived generated	27.64%	60.62%

**Table 2:** Result of tassel classification.

Number of training samples	Number of appended samples	Totalsamples = training + appended	Number of testing samples	Testing accuracy
87 (50%)	0	87	88 (50%)	0.94
87 (50%)	43 (25%)	130	88 (50%)	0.97
87 (50%)	87 (50%)	174	88 (50%)	0.98
87 (50%)	131 (75%)	218	88 (50%)	0.98
